# Deficits in Category Learning in Older Adults: Rule-Based Versus Clustering Accounts

**DOI:** 10.1037/pag0000183

**Published:** 2017-08

**Authors:** Stephen P. Badham, Adam N. Sanborn, Elizabeth A. Maylor

**Affiliations:** 1 Department of Psychology, Nottingham Trent University; 2 Department of Psychology, University of Warwick

**Keywords:** aging, categorization, learning, task complexity, clustering

## Abstract

Memory research has long been one of the key areas of investigation for cognitive aging researchers but only in the last decade or so has categorization been used to understand age differences in cognition. Categorization tasks focus more heavily on the grouping and organization of items in memory, and often on the process of learning relationships through trial and error. Categorization studies allow researchers to more accurately characterize age differences in cognition: whether older adults show declines in the way in which they represent categories with simple rules or declines in representing categories by similarity to past examples. In the current study, young and older adults participated in a set of classic category learning problems, which allowed us to distinguish between three hypotheses: (a) rule-complexity: categories were represented exclusively with rules and older adults had differential difficulty when more complex rules were required, (b) rule-specific: categories could be represented either by rules or by similarity, and there were age deficits in using rules, and (c) clustering: similarity was mainly used and older adults constructed a less-detailed representation by lumping more items into fewer clusters. The ordinal levels of performance across different conditions argued against rule-complexity, as older adults showed greater deficits on less complex categories. The data also provided evidence against rule-specificity, as single-dimensional rules could not explain age declines. Instead, computational modeling of the data indicated that older adults utilized fewer conceptual clusters of items in memory than did young adults.

Categorization is the process of grouping and organizing sensory information and draws upon many constructs in cognitive science including learning, decision making, reasoning and attention (Pothos & Wills, 2011). Understanding how individuals form categories from patterns in the environment is central to human learning (Feldman, 2000) and is relevant to a variety of circumstances in everyday life: Is it high or low fat? Are their policies left or right wing? Will this medication raise or lower blood pressure?

Surprisingly, given the extensive research into age differences in memory (e.g., Naveh-Benjamin & Ohta, 2012), there has been far less research into how young and older adults differ in the learning of categorical information (cf. Maintenant, Blaye, & Paour, 2011). Categorization research has the potential to deliver new insight into age-related changes in memory because these tasks can involve precise manipulations of the structure of categories to reveal the underlying representation. Therefore, categorization tasks can better assess the details of learning and the interference between competing items in memory than can most memory tasks.

A main point of contention in category learning is whether individuals are using rules or similarity to make their judgments. Rule-based approaches classically assume that there is a set of features that describe a category and a new stimulus is either entirely a category member or not (Bourne, 1970; Bruner, Goodnow, & Austin, 1986; Nosofsky, Palmeri, & McKinley, 1994). In contrast, similarity-based approaches assume that a new stimulus is compared either directly to exemplars experienced in the past, or to a single prototype of these past examples, producing a graded category membership (Medin & Schaffer, 1978; Nosofsky, 1986; Reed, 1972). The most flexible similarity-based approach is clustering, which because it clusters past exemplars into multiple prototypes, can produce representations that match exemplar models, prototype models, or anywhere in between (Anderson, 1991; Love, Medin, & Gureckis, 2004; Rosseel, 2002; Vanpaemel & Storms, 2008).

Initial conceptions of categorization were rule-based, but following theoretical and empirical arguments for graded category membership, similarity approaches became standard (Rosch, 1973; Wittgenstein, 1953). Later research leveraged the strengths of both rule-based and similarity-based categorization, through the development of hybrid models that have both an explicit rule-based system and an implicit similarity-based system (Ashby, Alfonso-Reese, Turken, & Waldron, 1998). Although there are empirical effects in category learning that point to both rule-based and similarity-based representations, it is possible that deficits in category learning in older adults are just of one type. Therefore, our question is: are age deficits best described as deficits in rule-based categorization or as deficits in similarity-based categorization?

Investigations into categorization deficits in older adults have compared young and older adults across various category structures to determine where the deficits for older adults lie, exploring both rule-based and clustering accounts. One rule-inspired hypothesis is that older adults are differentially worse at more complex categories (Cerella, Poon, & Williams, 1980), which we will term **rule-complexity**. For example, in Racine, Barch, Braver, and Noelle (2006) one category was composed of examples lying at the extremes of the space of possible continuous-feature stimuli, while the other was composed of examples lying in the middle of the space of possible stimuli. Participants were told what rule to follow, where category membership was defined by either a two- (low complexity) or three- (high complexity) part conjunctive rule. Racine et al. found that older adults performed differentially worse on the categories defined by more complex rules. Other categorization studies have supported the rule-complexity hypothesis by showing that as the task becomes more difficult, age-related deficits increase (so long as floor/ceiling effects are avoided). Additionally, studies involving functional relations (that are similar to categorization tasks in that participants must learn rules linking stimuli to responses) demonstrate greater age-related deficits for more complex relations, such as inverse (Griego & Kliegel, 2007) and multiplicative (Chasseigne, Lafon, & Mullet, 2002) relations.

A different rule-inspired hypothesis for age deficits follows from the model COVIS (Ashby et al., 1998). COVIS is a hybrid model consisting of two systems: an explicit system that can learn simple rules and an implicit system that can be considered similarity-based. It has been argued that categorization deficits in older adults (Rabi & Minda, 2016) and children (Minda, Desroches, & Church, 2008), relative to young adults, are larger for complex rule-based categorization tasks compared with implicit categorization tasks. Likewise, when increasing the number of irrelevant dimensions in a rule-based categorization task, Filoteo, Maddox, Ing, Zizak, and Song (2005) found a trend for older participants to perform differentially worse. Older adults may therefore have difficulty with explicit, rule-based categories that are arguably more reliant on effortful processing. Age-related memory deficits are generally reduced or absent for implicit tests of memory (La Voie & Light, 1994; Light, Prull, La Voie, & Healy, 2000), where effortful strategic encoding and retrieval processes are not required. Furthermore, age deficits in executive prefrontal processing (West, 1996) of rules have been used to describe older adults’ poor performance at the Wisconsin Card Sorting Test (Rhodes, 2004). Therefore, it seems that a dual-system account such as COVIS could explain age deficits in categorization in only its rule-based system but not the implicit system, a hypothesis we term **rule-specificity**. However, other researchers have shown the contrary effect: a larger age deficit in the implicit system than in the rule-based system (e.g., Filoteo & Maddox, 2004; Mata, von Helversen, Karlsson, & Cüpper, 2012).

In contrast to these rule-based accounts is the possibility that age deficits are implicit, and particularly the notion that older adults may not generate as detailed an implicit category representation as do young adults (Love & Gureckis, 2007), a hypothesis we call **clustering**. The assumption behind this hypothesis is that people use multiple prototypes (e.g., clusters) to represent categories, and the more clusters that are used the more detailed the category representation can be. Studies have shown that older adults can construct simple prototype representations as well as can young adults, but do not represent complex categories with as much detail as do young adults (Hess, 1982; Hess & Slaughter, 1986). Also, older adults have poorer memory for category members that are exceptions to rules (Davis, Love, & Maddox, 2012; Love & Gureckis, 2007). For example, Davis et al. (2012) showed participants images of beetles that were categorized into two groups. The features of the beetles were arranged such that the majority of beetles in one group would possess a given feature (e.g., thick legs) but a small subset would have the opposite feature (e.g., thin legs—an exception to the rule). Older adults showed a deficit relative to young adults when categorizing these exception stimuli. This can be explained as older adults constructing fewer clusters than young adults to represent categories.

In summary, we have identified three hypotheses related to age differences in categorization: (a) rule-complexity: differential difficulty with category structures defined by more complex rules in a rule-only categorization model, (b) rule-specific: age deficits in the use of explicit rule-based but not implicit systems of a hybrid model, and (c) clustering: a tendency for older adults to construct fewer clusters in similarity-based categories. These explanations are difficult to tease apart: they can imitate one another quite closely as more complex categories also generally require both more complex rules and more clusters to be represented accurately. Researchers have only begun to test these accounts against one another: Rabi and Minda (2016) compared the two rule-based accounts and found evidence to support rule-specificity over rule-complexity. The key evidence was smaller age deficits in a more complex categorization task compared with a less complex categorization task. However, this study did not rule out age deficits attributable to clustering. The current study aimed to replicate and further explore this key empirical finding to determine if a rule-specificity account of deficits is plausible, and also to establish if the empirical age deficits could be explained better with a clustering account.

## The Current Study

**Figure 1 fig1:**
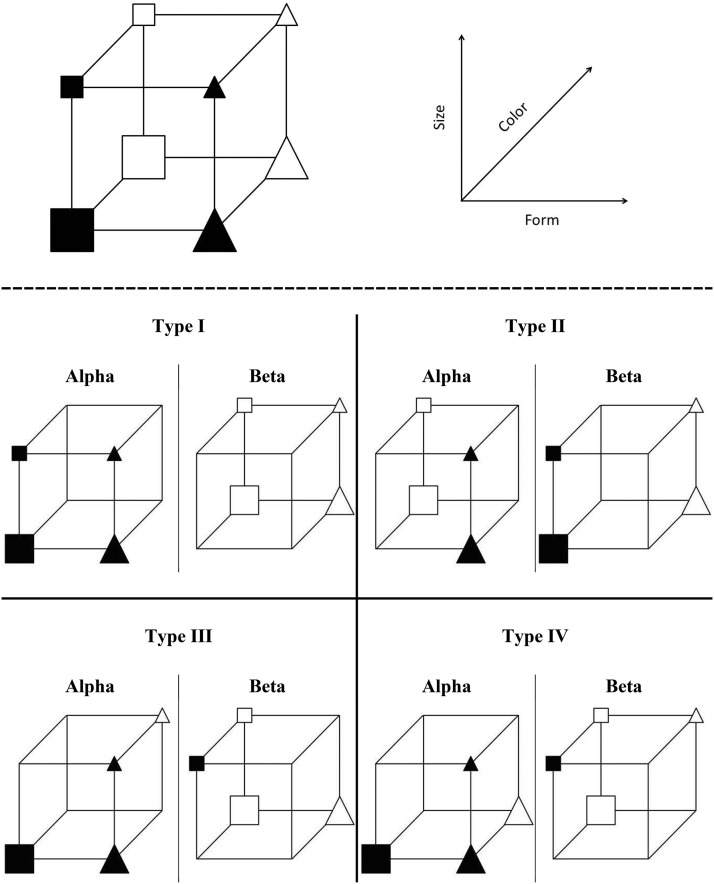
Top: Stimuli could vary along three dimensions (size, color, and form). Bottom: Examples of category membership for the eight shapes organized into two groups (α and β) for the four categorization tasks (Types I to IV) used in the study.

Here, we compare the rule-complexity, rule-specificity, and clustering hypotheses of age deficits against one another using a seminal paradigm from the categorization literature, the category learning problems of Shepard, Hovland, and Jenkins (1961). In this task, participants learn to place a series of eight geometric images into two categories across a series of learning blocks. The eight images were formed by factorial combinations of three binary dimensions (see top panel of Figure 1), which were form (square/triangle), color (black/white), and size (large/small). Four of the shapes were assigned to an “α” group and four to a “β” group.[Fig-anchor fig1]

Shepard et al. (1961) identified six meaningfully distinct ways to form two groups of four stimuli from the set of eight geometric images (Types I, II, III, IV, V, and VI). These groupings are based upon categorization rules of varying complexity and Types I to IV were used in the current study (see bottom panel of Figure 1). Type I is the simplest condition where a single dimension defines category membership (e.g., all the black images are in the α category and all the white images are in the β category) and the other dimensions (e.g., size and form) are irrelevant. Type II defines category membership by two dimensions (e.g., black triangles and white squares are in the α group) with one irrelevant dimension (e.g., size). Type III uses all three dimensions to define category membership and categories are defined by a rule with an exception (e.g., all the black objects are in the α group apart from the small black square). Type IV also uses all three dimensions and all category members share the majority of their features with other category members (e.g., most of the large, black, and triangular shapes are in the α group). Types III and IV seem similar and indeed often lead to similar levels of performance (e.g., Shepard et al., 1961) but one key difference is that participants can respond with 75% accuracy by paying attention to *any* single dimension for Type IV but can only achieve 75% accuracy in Type III with a single dimension for two out of the three dimensions (e.g., responding on the basis of color or form alone for Type III in Figure 1 would yield 75% accuracy but size would yield 50% accuracy).

In young adults, performance generally decreases from Type I to Type IV (Type I > Type II > Type III = Type IV; Kurtz, Levering, Stanton, Romero, & Morris, 2013; Nosofsky, Gluck, Palmeri, McKinley, & Glauthier, 1994; Shepard et al., 1961). For the rule-complexity hypothesis (Cerella et al., 1980), the prediction is simply that—unless there are floor/ceiling effects—age differences will follow this same pattern, that is, increasing age differences from Type I to Type IV. This hypothesis about rule-complexity based on learning difficulty is bolstered by formal mathematical analyses of the complexity of the rules needed to learn Types I–IV. Feldman (2000) introduced an explicit Boolean complexity measure of the Shepard et al. (1961) types, finding that this formal measure of complexity corresponded fairly closely to learning difficulty. Although there is some disagreement about the relative difficulty of Type III, Boolean complexity and various other measures of complexity agree that Type IV is more complex than Type II that itself is more complex than Type I (Goodman, Tenenbaum, Feldman, & Griffiths, 2008; Vigo, 2006, 2009). Therefore, both mathematical and behavioral accounts of complexity would predict greater age differences for Type IV than for Type II and greater age differences for Type II than for Type I.

Rabi and Minda (2016) found age differences that clearly went against the predictions of rule-complexity. Whereas young adults showed better performance on Type II than Type IV, older adults showed the opposite: their Type IV performance exceeded their Type II performance. The deficit in Type II was taken as evidence of rule-specificity in age deficits. It was argued that older adults were generally not able to use multidimensional rules, because of their poor overall performance on Type II. Also it was argued that older adults were unable to transition to the more flexible implicit system, and so “applied single-dimensional rules during Type II learning, but frequently switched rules during the course of the task to avoid negative feedback” (p. 194). Thus, we formulate the rule-specificity hypothesis to mean that older adults cannot use multidimensional rules, and must use either single-dimensional rules or their intact implicit system instead. This rule-specificity account was bolstered by an association between backward digit span and Type II performance, plausibly tying complex rule-based categorization to working memory capacity. Rule-specificity was also suggested to explain the reliable deficit older adults showed in Type IV performance: this was potentially a result of older adults following simple rules in Type IV rather than switching over to the more flexible implicit similarity-based system, as young adults do (e.g., Maddox et al., 2010).

Explanations based on deficits in rule use, however, are not the only kind of explanation for these age deficits. A different hypothesis was investigated by Davis et al. (2012) who found that older participants struggled much more with learning the exceptions to rules (summarized earlier). They proposed a clustering account of their results, that is, items were grouped into clusters, each of which is represented as a prototype of the items in a cluster. Clustering explanations essentially represent a category by multiple prototypes, which interpolates between the extremes of single prototype models and exemplar models (that represent all of the previously experienced items individually). Davis et al. supposed that older adults have more difficulty in constructing new clusters of items in memory, meaning that categories are represented more coarsely by older adults (see also Love & Gureckis, 2007). Their argument was bolstered by fitting their data with a clustering model of categorization, the Rational Model of Categorization (RMC; Anderson, 1991), and showing that the parameters indicated that older adults did not construct as many clusters as did young adults.

Intuitively, a clustering account could also explain the pattern of age deficits found by Rabi and Minda (2016). Types I and IV can both be represented well by a single cluster or prototype per category because the two categories in these tasks are linearly separable: a straight plane can be placed in the space of stimuli in Figure 1 for these two tasks that perfectly separates the two categories. In contrast, representing each category in Type II with a single cluster would be a catastrophe: because of the symmetric arrangement of the stimuli in each category, the prototype of each cluster would be exactly in the middle of the cube of stimuli, and so the inferred categories are indistinguishable and performance would be at chance. Thus, the number of clusters is more critical in Type II than in Type I or Type IV, and so if older adults have greater difficulty constructing more clusters, then larger age differences are expected in Type II compared with Types I or IV, matching the empirical results.

Although it is intuitive that a clustering account can explain age deficits, we cannot know whether rule-specificity or clustering deficits better match human behavior until we evaluate them against data. We collected our own data in the Shepard et al. (1961) tasks, including Type III in addition to Types I, II, and IV, which first allowed us to determine if the pattern of age deficits replicated. Type III provides another benchmark against which to evaluate Types I, II, and IV, and an opportunity to see if Types III and IV are also equally difficult for older adults, as seen in young adults and as many complexity approaches predict. Using these new data, we then evaluated the plausibility of the idea that older adults were using single-dimensional rules, using a variety of measures. We finally fit the RMC to the trial-by-trial data to see if a clustering account could quantitatively match the data.

## Method

### Design

Young and older adults learned to categorize eight shapes into two groups. Each participant completed four conditions (Types I to IV) where group membership was determined by separate rules as outlined in the introduction.

### Participants

Forty-eight young adults (42 female) aged 18–21 years (*M* = 19.3, *SD* = 0.7) and 48 healthy older adults (32 female) aged 60–87 years (*M* = 74.7, *SD* = 5.6) took part in the experiment. Ten of the older adults were in their 60s, 30 in their 70s, and eight in their 80s, with all except four aged 66–83. Young participants were recruited from the University of Warwick and received course credit. Older participants were active members of our Age Study Panel who were visited in their own homes and received £5 ($7); their self-rated eyesight, hearing, and general health averaged 4.1, 4.0, and 4.0 (equivalent to “good”), respectively, on a 5-point scale (1 = *very poor* to 5 = *very good*). Participants were recruited in two batches (though all were tested within a 7-week period, January through March, 2015), and the statistical implications of this are discussed in the results. All participants provided written informed consent, and the study was approved by the University of Warwick’s Humanities and Social Sciences Research Ethics Committee.

Young and older participants did not show evidence of a difference in their years of education, *t*(53.87) = 1.88, *BF*_*10*_ = 1.01^1^ (*M*_young_ = 14.01, *SD*_young_ = 0.93; *M*_older_ = 14.97, *SD*_older_ = 3.41). To assess cognitive functioning, participants completed the Digit Symbol Substitution test from the Wechsler Adult Intelligence Scale—Revised (Wechsler, 1981) as a measure of processing speed, and the multiple choice part of the Mill Hill vocabulary test (Raven, Raven, & Court, 1988) as a measure of crystallized intelligence. The results were consistent with the literature (e.g., Salthouse, 2010): young adults performed better than older adults at the speed task, *t*(94) = 10.98, *BF*_*10*_ = 2.42 × 10^15^ (*M*_young_ = 74.63, *SD*_young_ = 10.04; *M*_older_ = 51.96, *SD*_older_ = 10.19), and older adults performed better than young adults at the vocabulary task, *t*(94) = 8.99, *BF*_*10*_ = 1.95 × 10^11^ (*M*_young_ = 16.83, *SD*_young_ = 3.47; *M*_older_ = 23.67, *SD*_older_ = 3.96).

### Materials

Images of eight geometric shapes were constructed for use in the experiment. Large images had a base of width 250 pixels and small images had a base of width 125 pixels, corresponding to widths of approximately eight and four degrees of viewing angle on screen, respectively. Triangles were equilateral and both square and triangle image bases were horizontal. Images were presented in black or white on a midgray background.

#### Counterbalancing

The four conditions were within participants so this resulted in 24 possible test orders for Types I to IV. Additionally, each condition had several permutations (e.g., Type I had three permutations because category membership could be defined by color, form, or size). Types II, III, and IV had 3, 12, and 4 permutations, respectively. Twenty-four versions of the experiment were created (one for each test order) and the permutations of each type were randomly assigned to each version such that each permutation was used equally across the experiment (for simplification, Type III was reduced to 3 permutations by assigning all but one dimension randomly). Category memberships α and β were also randomly determined. These 24 versions of the experiment were then used four times (twice with young and twice with older adults).

### Procedure

Participants were initially shown rule-based instructions taken verbatim from Kurtz et al. (2013) who found that such instructions are more likely to yield the typical Type II advantage (relative to Types III and IV) shown in the literature. These instructions encourage participants to “learn a rule that allows [them] to tell whether each example belongs in the alpha or beta category” (Kurtz et al., 2013, p. 6). Participants were then shown a single screen containing all eight shapes (in no particular arrangement, and without any category information) so that they could clearly see the differences between the shapes. They were informed that these were all of the shapes that would be used in the experiment. Following this, they commenced the first condition of the experiment.

In each trial, an image was presented centrally on the screen. Participants were initially required to guess if it belonged in the α or β category by pressing the keys “F” and “J” on the computer keyboard, which were relabeled “Alpha” and “Beta,” respectively (the words Alpha and Beta were also displayed in the bottom left and right corners of the screen, respectively). The image remained on screen until a response was made, then after 500 ms of blank screen, feedback was provided. The image reappeared on screen and either “Correct!” appeared above it in green or “Incorrect!” in red. For both feedback options, below the feedback image appeared the correct response in blue, for example, “Answer = Alpha.” The feedback remained on screen until the participant pressed the spacebar, then a further 500 ms of blank screen was displayed before the next trial.

In the first two blocks, all eight shapes were presented in the first half of the block and then again in the second half. This limited the possibility of the same shape appearing in adjacent trials. In subsequent blocks, the eight shapes were presented twice in each block of 16 trials without any constraints. This ordering replicates the original Shepard et al. (1961) study. Participants completed the task for six blocks (96 trials) or until they reached a criterion of perfect performance in two consecutive blocks. Once a condition was complete, a message on screen indicated that “a new rule [would] determine which images belong to each category.” Participants could rest between conditions as they wished. The experiment continued until the participant had completed all four categorization conditions.

## Results

During our data collection process, we found interesting trends (qualitatively identical to those we report below) after testing 48 participants (i.e., 24 young and 24 older), but the key comparison (namely, the age by condition interaction) did not reach the standard value for statistical significance. Therefore, we tested an additional 48 participants and stopped our experiment at that point. This stopping rule invalidates the *p* values calculated using standard null hypothesis significance (e.g., Wagenmakers, 2007), so we report test statistics and effect sizes without the *p* values. Instead we report Bayes factors, which provide a valid measure of the evidence provided by the data even when the rule for stopping data collection depends on the results of a test (Rouder, 2014). This measure even provides strong guarantees about how much an experimenter can influence the statistical results, in particular when finding evidence that favors the alternative hypothesis (Sanborn & Hills, 2014).

Standard null hypothesis significance tests assess the probability of a test statistic arising from the null hypothesis, limiting researchers to only evaluating the plausibility of the null, and leaving them in an awkward position if there is not enough evidence to reject the null. In contrast, Bayesian methods explicitly compare the probability of the null and alternative hypotheses on even ground, so that evidence can be found in favor of the alternative hypothesis, the null hypothesis, or neither (Gallistel, 2009; Rouder, Speckman, Sun, Morey, & Iverson, 2009). A common Bayesian measure of evidence is the Bayes factor (*BF*_*10*_; Kass & Raftery, 1995) that provides an odds ratio for the alternative/null hypotheses (values <1 favor the null hypothesis and values >1 favor the alternative hypothesis). For example, a *BF*_*10*_ of 2.5 would indicate that the alternative hypothesis is 2.5 times more likely than the null and a *BF*_*10*_ of 0.40 would indicate the converse (see Jarosz & Wiley, 2014). Associating labels with these values is arbitrary, but in past work labels such as “substantial,” “strong,” and “decisive” have been associated with Bayes factors of 3, 10, and 100, respectively (Wetzels et al., 2011). These Bayes factors were calculated using the JASP computer software (Love et al., 2015). All *t* tests are two-tailed using the standard Cauchy prior width of 0.707. The Bayesian analyses of variance (ANOVAs) construct a model for each of the possible combinations of terms and we report BF_inclusion_ for each term because it gives a summary of the evidence for including that term in the models.

**Figure 2 fig2:**
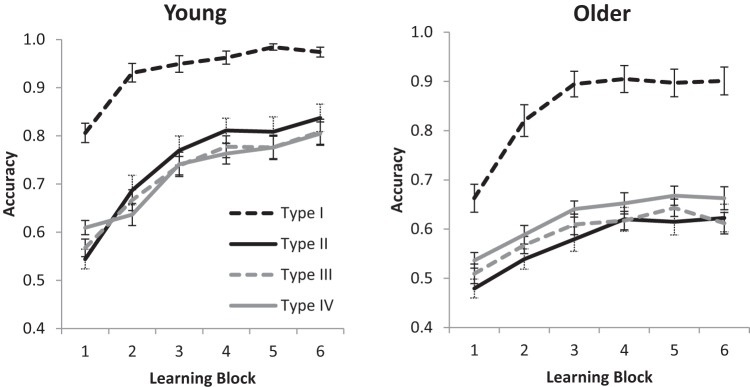
Accuracy for young and older adults learning categorization Types I, II, III, and IV across six learning blocks (16 trials per block). Error bars are ±1 *SE*.

**Figure 3 fig3:**
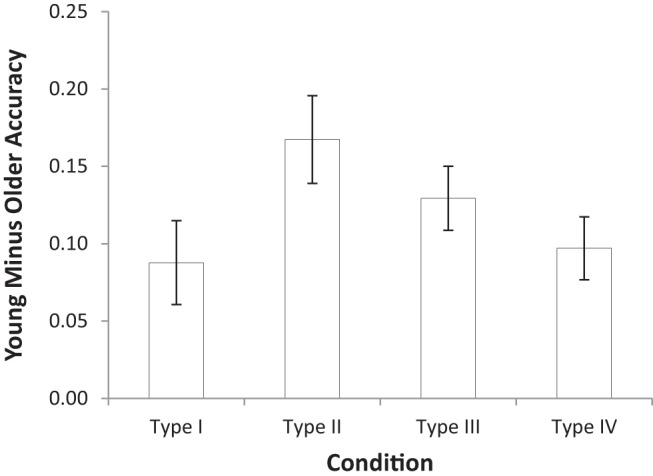
Magnitude of age deficits in learning for the different experimental conditions Types I to IV. Data are averaged across all six blocks. Error bars are ±1 *SE*.

For the accuracy data, where a condition was terminated early because of a participant reaching criterion, 100% accuracy was assumed for all subsequent uncompleted blocks (as is typical with this paradigm: e.g., Kurtz et al., 2013; Nosofsky, Gluck, et al., 1994). Figure 2 shows the overall means for Blocks 1–6, while Figure 3 shows overall age differences, for Types I–IV. Performance accuracy was entered into a 2 (Age: young, older) × 4 (Condition: Types I to IV) × 6 (Block: 1–6) repeated measures ANOVA. Young adults were more accurate than older adults, *F*(1, 94) = 48.86, *MSE* = 0.17, η_p_^2^ = .34, *BF*_*10*_ = 3.16 × 10^12^. There was a main effect of condition,^2^ *F*(2.59, 243.10) = 129.83, *MSE* = 0.07, η_p_^2^ = .58, *BF*_*10*_ > 10^12^, with Type I learned better than all other conditions (performance on Types II to IV is investigated further below). A main effect of block showed that performance improved over time, *F*(3.31, 311.55) = 138.91, *MSE* = 0.02, η_p_^2^ = .60, *BF*_*10*_ > 10^12^. Age interacted with condition, *F*(2.59, 243.1) = 3.26, *MSE* = 0.07, η_p_^2^ = .03, *BF*_*10*_ = 7651, but there was no evidence that it interacted with block, *F*(3.31, 311.55) = 4.02, *MSE* = 0.02, η_p_^2^ = .04, *BF*_*10*_ = 0.88. There was sizable evidence against the three-way interaction between age, condition, and block, *F*(10.64, 999.82) = 3.31, *MSE* = 0.02, η_p_^2^ = .03, *BF*_*10*_ = 4 × 10^−3^. As can be seen in Figure 2, the young adults’ Type I performance was near ceiling, which could potentially be driving the age by condition interaction (see Figure 3). [Fig-anchor fig2][Fig-anchor fig3]

To investigate potential age interactions without ceiling performance, the above ANOVA was repeated but with Type I excluded from the condition factor. Young adults performed better than older adults, *F*(1, 94) = 53.78, *MSE* = 0.14, η_p_^2^ = .36, *BF*_*10*_ = 1.33 × 10^12^, there was evidence for a main effect of condition, *F* < 1, *BF*_*10*_ = 3.62, and accuracy improved across blocks, *F*(3.46, 324.82) = 87.48, *MSE* = 0.03, η_p_^2^ = .48, *BF*_*10*_ > 10^12^. There was an age by block interaction, *F*(3.46, 324.82) = 7.61, *MSE* = 0.03, η_p_^2^ = .08, *BF*_*10*_ = 1.02 × 10^4^, because of slower learning in older adults. More important, the age by condition interaction remained, *F*(1.70, 159.94) = 3.61, *MSE* = 0.06, η_p_^2^ = .04, *BF*_*10*_ = 20.50, confirming the different age-related deficits between Types II–IV evident in Figure 3; this interaction is investigated further below. There was evidence against the other interactions in the analysis (Condition × Block, *F* < 1.36, *BF*_*10*_ = 2 × 10^−3^, Age × Condition × Block, *F* < 1, *BF*_*10*_ = 2.65 × 10^−5^).

To interpret the above age by condition interaction, the condition by block (3 × 6) ANOVA was run separately for young and older adults. Older adults had a main effect of condition, *F*(1.78, 83.51) = 4.83, *MSE* = 0.04, η_p_^2^ = .09, *BF*_*10*_ = 186.6, but there was evidence that young adults did not, *F* < 1, *BF*_*10*_ = 7.5 × 10^−2^. *T* tests (collapsed across blocks) revealed that older adults performed best at Type IV, *M* = 0.62, *SD* = 0.08, which was better than Type II, *M* = 0.57, *SD* = 0.11, *t*(47) = 3.10, *BF*_*10*_ = 10.04, and possibly better than Type III, *M* = 0.59, *SD* = 0.08, *t*(47) = 2.36, *BF*_*10*_ = 1.92 (whereas Type II and III performance appeared the same, *t* < 1, *BF*_*10*_ = 0.24). Numerically, young adults performed best at Type II, *M* = 0.74, *SD* = 0.16, but there was evidence that performance did not differ from that in Type III, *M* = 0.72, *SD* = 0.12, *t*(47) < 1, *BF*_*10*_ = 0.229, and Type IV, *M* = 0.72, *SD* = 0.11 *t*(47) < 1, *BF*_*10*_ = 0.232. There was also evidence that performance did not differ between Type III and Type IV, *t*(47) < 1, *BF*_*10*_ = 0.157.

### Testing for Rule Use

The rule-specificity hypothesis is that older adults show deficits in the rule-based system, but have an intact implicit system. Because older adults perform worse across all four types, the rule specificity hypothesis implies that all of these declines are because of worse rule-based categorization. In particular, Rabi and Minda (2016) hypothesized that older adults are only rarely able to use conjunctive or disjunctive rules and instead must rely on single-dimensional rules. This can explain the superior performance that older adults demonstrated on Type IV versus Type II: any single-dimensional rule would result in 75% accuracy for Type IV, but result in 50% accuracy for Type II.

**Figure 4 fig4:**
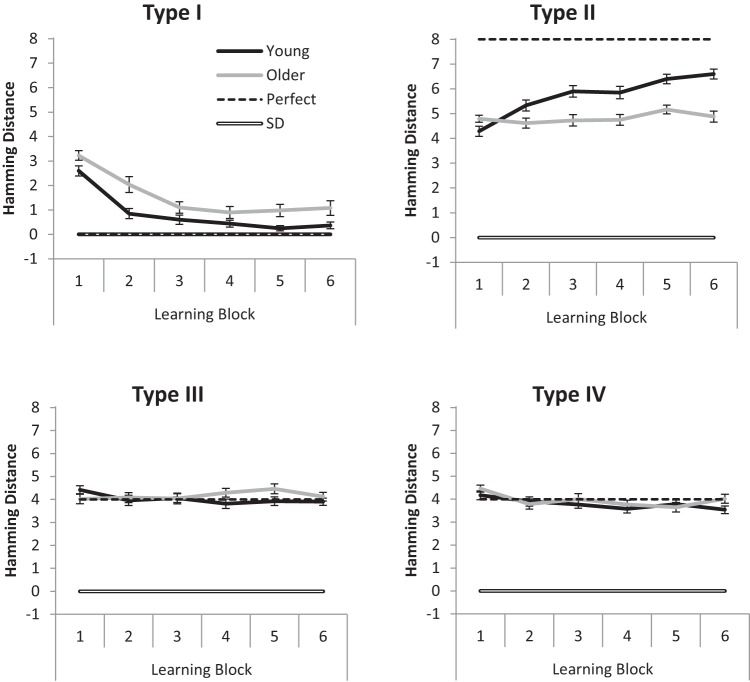
Hamming distance for young and older adults across learning Blocks 1–6. The dashed line indicates the Hamming distance that would occur if participants were responding with 100% accuracy for each type, though this is necessary and not sufficient to produce perfect performance: matching this distance does not imply 100% accuracy. The hollow line indicates the distance corresponding to single-dimensional rule use (SD). Error bars are ±1 *SE*.

We first investigated whether conjunctive and disjunctive, or single-dimensional rules were used by looking at the consistency with which individuals were adhering to these rules in each of the four problems. To do so, we created a measure that is diagnostic as to whether single-dimensional rules are being used. First, we computed the number of mismatches (i.e., Hamming distance) between the responses in each block and the responses that would have been made using each of the three possible single-dimensional rules. Then the minimum of the three Hamming distances in each block was taken as the measure of adherence to the closest single-dimensional rule. The result is a score for each individual in each block, and the mean scores for the two age groups over the blocks are shown in Figure 4. Here, perfect performance would result in (minimum) Hamming distances of zero for Type I, eight for Type II, and four for both Types III and IV. If participants are consistently using a single-dimensional rule for any problem, then the Hamming distance will be zero.[Fig-anchor fig4]

For each type, a 2 (Age: young, older) × 6 (Block: 1–6) repeated measures ANOVA was conducted. For Type I, older adults had larger Hamming distances than young adults, *F*(1, 94) = 7.57, *MSE* = 9.36, η_p_^2^ = .08, *BF*_*10*_ = 4.55, and the Hamming distances decreased across blocks showing a trajectory toward the correct distance of zero, *F*(3.10, 291.20) = 81.31, *MSE* = 1.52, η_p_^2^ = .46, *BF*_*10*_ > 10^12^, with no interaction, *F*(3.10, 291.20) = 1.75, *MSE* = 9.36, η_p_^2^ = .02, *BF*_*10*_ = 0.534.

For Type II, older adults had smaller Hamming distances than young adults, *F*(1, 94) = 21.02, *MSE* = 5.63, η_p_^2^ = .18, *BF*_*10*_ = 1.42 × 10^7^, the Hamming distances increased across blocks, *F*(4.31, 405.15) = 15.30, *MSE* = 1.60, η_p_^2^ = .14, *BF*_*10*_ > 10^12^, and to a greater extent in young compared with older adults, *F*(4.31, 405.15) = 10.11, *MSE* = 1.60, η_p_^2^ = .10, *BF*_*10*_ = 2.79 × 10^9^. Note that post hoc tests revealed that young adults showed a trajectory toward the correct distance of eight across blocks, *F*(3.37, 158.49) = 25.16, *MSE* = 1.97, η_p_^2^ = .35, *BF*_*10*_ > 10^12^, but there was evidence that older adults remained constant across blocks, *F*(5, 235) = 1.17, *MSE* = 1.43, η_p_^2^ = .02, *BF*_*10*_ = 0.05. Thus, it appears that older adults were neither trending toward using single-dimensional rules consistently, nor trending toward using the correct multidimensional rules consistently. Their responses in Type II problems were stuck between these two extremes, and did not change across blocks.

For Type III, there was no effect of age, *F* < 1, *BF*_*10*_ = 0.153, the Hamming distances did not change across blocks, *F* < 1, *BF*_*10*_ = 0.003, and there was no interaction, *F*(5, 470) = 1.95, *MSE* = 1.47, η_p_^2^ = .02, *BF*_*10*_ = 6.93 × 10^−4^.

For Type IV, there was no effect of age, *F* = 1.00, *BF*_*10*_ = 0.161, the Hamming distances decreased across blocks, *F*(5, 470) = 3.52, *MSE* = 1.44, η_p_^2^ = .04, *BF*_*10*_ = 1.693, and there was no interaction, *F* < 1, *BF*_*10*_ = 0.018.

For Types III and IV, all participants were close to the Hamming distance that perfect performance would produce across all blocks. However, this only shows that their responses showed the right amount of deviation from single-dimensional rules—clearly the actual responding of both young and older adults was far from perfect for these two types (see Figure 2).

From the Hamming distance measures, older adults appear to be unable to learn the multidimensional rules required for Type II problems, and also do not appear to be using single-dimensional rules consistently instead. Of course, older adults may not be using single-dimensional rules consistently throughout a block as the Hamming distance measures, but instead are quickly switching between single-dimensional rules as they accumulate negative feedback (Ashby et al., 1998; Rabi & Minda, 2016). Fortunately, the Shepard et al. (1961) stimuli allow us to assess how often quick switches in single-dimensional rules are occurring by looking for consecutive trials in which the stimuli are maximally distant from one another (i.e., in Figure 1 pairs of stimuli that are in the opposite corners of the cube from one another). Looking at these consecutive trials (that make up 13% of all trials), participants who use the same single-dimensional rule in the two trials will always make two different responses, no matter which single-dimensional rule is used.

**Figure 5 fig5:**
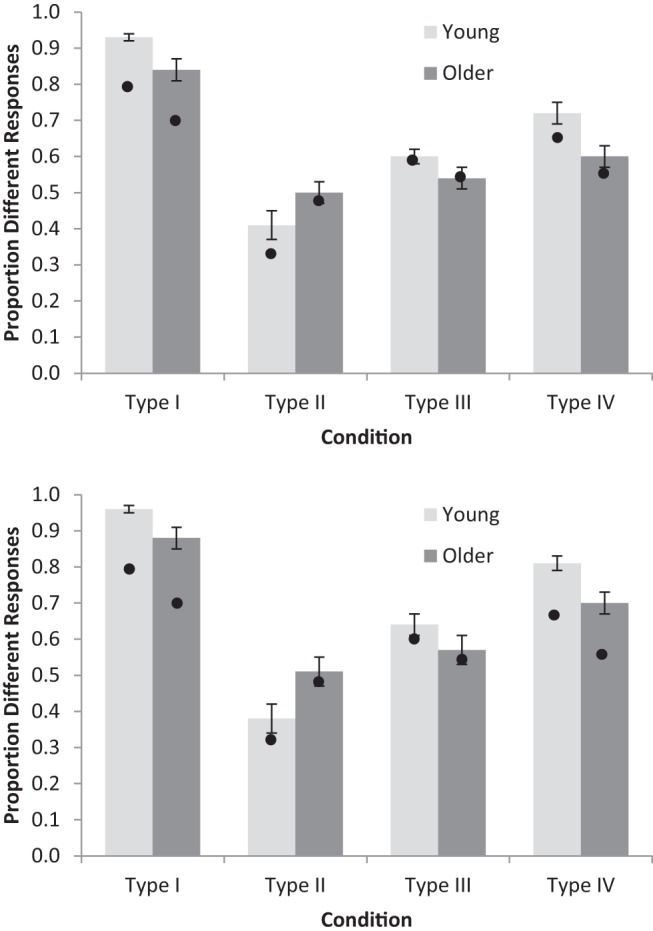
Proportion of different responses to all consecutive trials with maximally different stimuli (top panel), and to consecutive trials with maximally different stimuli where the previous response was correct (bottom panel). Error bars are ±1 *SE*. Black circles indicate predictions from the Rational Model of Categorization.

Figure 5 shows the proportion of trials on which young and older participants made the different responses to maximally different stimuli on consecutive trials, meaning that the two responses were consistent with using the same single-dimensional rule. The other types are included for completeness, but Type II is the most interesting task in this analysis because of the possibility that older adults are quickly switching between single-dimensional rules as they are unable to use multiple dimensional rules. In Type II only there is also a clear contrast between correct responding and consistent single-dimensional rule use: correct responding predicts a value near 0 while single-dimensional rule use predicts a value near 1. As shown in Figure 5, young adults make the same response more than half the time, while older adults make the same response almost exactly half the time (and only two older adults never made this response). Such a low percentage of different responses cannot result from consistent use of single-dimensional rules even across two consecutive trials; instead it looks most like randomly selecting a single-dimensional rule on each trial. What is particularly striking is that the proportion of different responses (indicating single-dimensional rule use) is only half, even when older adults made the correct response to the previous trial. This is notable because the COVIS explicit system, which is used as the basis for the rule-specificity account, assumes that a rule will always be used again on the next trial if it is successful (Ashby et al., 1998).[Fig-anchor fig5]

### Interim Summary

In brief, young adults performed better than older adults at the categorization tasks and the two age groups had qualitatively different patterns of performance: For young adults, our data replicated the traditional pattern of accuracy (Type I > Type II > Type III = Type IV; e.g., Shepard et al., 1961). However, older adults showed superior performance in Type IV compared with Type II. These age differences were similar to those found by Rabi and Minda (2016) who hypothesized that older adults’ performance was driven by increased reliance on single-dimensional rules during learning. In the current study, statistical tests of single-dimensional rule use did not support this hypothesis.

### Model-Based Analysis

We presented the intuition above that constructing fewer clusters in the RMC (Anderson, 1991) would result in the observed age deficits. To verify that young and older adults did construct different numbers of clusters and that it could produce the same pattern of age deficits, we fit the RMC to the data. The RMC is a model that infers which items belong together in clusters, based on both their physical features and their category labels. In this model, the category label is treated as just another feature, so it is possible that items from two separate categories will be placed in the same cluster. When making category judgments, the RMC first finds the probability that the new item comes from each of the clusters (including the possibility that the item belongs in a new cluster) and then weights the prediction of each cluster/level of the category label by these probabilities.

The RMC used three parameters in its original formulation: a coupling parameter, *c*, a physical salience parameter, *s*_*P*_, and a label salience parameter, *s*_*L*_. The coupling parameter controls the prior probability of the number of clusters. A high coupling parameter means there will be fewer clusters, whereas a low coupling parameter means there will be more clusters. The two salience parameters control how “pure” each of the clusters are along the physical (e.g., size, form, and color) or label features, with lower values meaning that each cluster is more likely to contain only a single value of each feature (e.g., this cluster will only have triangles or only squares). For the label salience parameter, a low value means that it is less likely that two items from different categories will be placed in the same cluster. The RMC is also often augmented by a determinism parameter, *r*, which acts to bring response probabilities either closer to chance for low *r* or closer to deterministic performance for high *r* (Nosofsky, Gluck, et al., 1994). Full details of the RMC are given in Appendix A.

To investigate which parameters were responsible for the differences between the age groups, we created a set of 16 models. Every model was fit using the same parameters for all participants within an age group, but the different models allowed for different sets of parameters to differ between groups. A description of each model along with several measures of how well each fit the data is shown in Table 1. For all of these measures, a lower value indicates a better model. The negative log likelihood was computed across all participants and only measures the fit to the data, while Akaike’s Information Criterion (AIC) and Bayesian Information Criterion (BIC) adjust the overall negative log likelihood with penalties for model complexity. We also converted AIC and BIC values into the more interpretable AIC and BIC weights, which approximate the probability of each model given the data, assuming the models are equally likely before the experiment began (Akaike, 1978; Kass & Raftery, 1995; Wagenmakers & Farrell, 2004).[Table-anchor tbl1]

**Figure 6 fig6:**
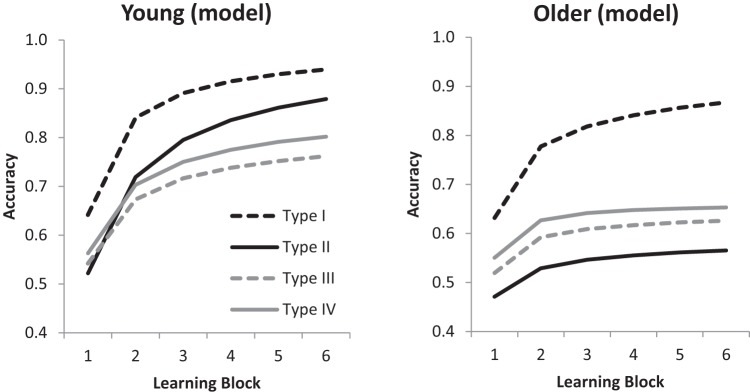
Model predictions for young and older adults learning categorization Types I, II, III, and IV across six learning blocks.

Using both AIC and BIC weights, the best model was clearly Model 14, which allowed for three of the four parameters to differ between young and older adults: *s*_*P*_, *c*, and *r*. The performances of young and older adults predicted by this model are shown in Figure 6, and they generally match the human data well. The main discrepancy is that within each age group the model did not learn Type I tasks as quickly as participants did, but the overall accuracy predicted by the best-fitting parameters matched the ordering of accuracy on the problem types for each age group.[Fig-anchor fig6]

**Figure 7 fig7:**
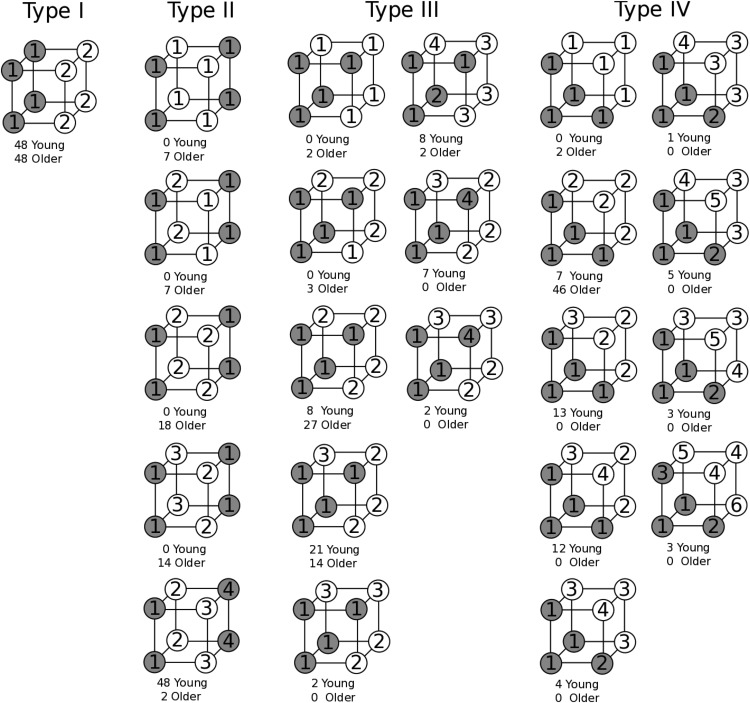
Visualization of how young and older adults clustered the items for Types I–IV. The plots underneath each type display the different ways the problem was clustered across participants. Within each plot, the three dimensions represent the three different physical dimensions, though the dimension identities have been ignored and cluster assignments renumbered to minimize the variety of different patterns. Gray and white circles indicate the feedback given to the items and the numbers within each circle label the cluster to which that item has been assigned. Text underneath each plot gives the number of young and older adults who used that set of clusters for that problem.

The best-fitting values of Model 14’s parameters are shown in Table 2. Older adults had a higher best-fitting coupling parameter than did young adults, implying that they formed fewer clusters. However, unlike Davis et al. (2012), we allowed the physical and label salience parameters to vary, as these parameters can also affect the clustering of the stimuli. A more direct view of how young and older adults clustered the stimuli can be obtained by looking at assignments of items to clusters made by the model. We found that the different orders in which the trials were presented led to variability in the clusters formed across individuals with the same parameters. In Figure 7, we show the assignments made by the model for the last block of stimuli in the experiment. Whereas young and older adults both used two clusters for Type I, the model indicates that older adults were more likely to use fewer clusters to represent each of Types II–IV. Overall, older adults were not using as many clusters as were young adults.[Table-anchor tbl2][Fig-anchor fig7]

The differences in parameters between young and older adults do not just impact how the items are clustered. These parameters also impact how a category judgment is made given a particular representation. Older adults had higher values of *c*, as well as lower values of *s*_*P*_ and *r*. For new items, the value of *c* controls the influence of the existing clusters relative to a new cluster that contains just the new item and has no label information. As a result, the higher value of *c* means that older adults have stronger category preferences than young adults given their representation. Relatedly, lower values of *s*_*P*_ for older adults mean that items will have a stronger match to clusters they belonged to in previous blocks, increasing the strength of category preferences. However, the lower value of *r* for older adults means that responses will be more stochastic and that the most likely category label will not be chosen as often.

To determine the overall impact of these parameter differences on how category labels are chosen for incoming items, we looked at what would happen if older adults clustered like older adults, but made choices like young adults. This was done to establish whether the predicted reversal in Type II and Type IV performance was because of the clustering of items or to the choice parameters. In essence we used different parameters at different stages of each trial: the young adults’ parameters were used when making a category label prediction, but after receiving feedback the older adults’ parameters were used to assign an item to a cluster. The impact of using the older adults’ choice parameters on accuracy can be seen in Table 3. If older adults behaved like young adults while predicting category labels, then they perform equivalently or slightly better than young adults for Type I (because young and older adults used the same clusters), and perform better but not as well as young adults for Types II–IV. More important, the performance on Type IV problems is still predicted to be more accurate than on Type II problems, which means that the clustering, rather than the choice parameters, is controlling relative performance for these two problem types for older adults.[Table-anchor tbl3]

Beyond the accuracy on Types I–IV, we also investigated what the best-fitting version of the RMC predicted for the statistics we developed to test for the presence of single-dimensional rules. Predicted Hamming distances were calculated by finding the expected minimum distance to the set of single-dimensional rules for each block based on the model predictions for each stimulus. The predicted distances matched the empirical distances well, with the exception that participants corresponded to single-dimensional rules in the Type I task better than the model predicted. The Pearson correlation between model predictions and empirical distances across all participants, types, and blocks was .95.

Figure 5 shows the RMC predictions for consecutive maximally different stimuli. For the lower panel (“previous response correct”), the trials selected were just the same trials selected in the analysis of the data. These model predictions show the same overall patterns as the human data, in particular the near 0.5 rate of different responding for older adults in the Type II task. The Pearson correlation between the model and data across age groups and tasks for all responses was .96, and for previous response correct the correlation was .97.

## Discussion

We investigated three hypotheses of the source of age differences in categorization in our experiment: rule-complexity, rule-specificity, and clustering. In line with Rabi and Minda (2016), our results supported an age-related reversal of performance: Type II task performance was reliably worse for older adults than Type IV task performance, but Type II performance was statistically the same (and numerically better) than Type IV for young adults.^3^ Because the rule-complexity hypothesis predicts that Type IV performance would be impacted more than Type II performance for older adults, this effect serves as strong evidence against a rule-complexity explanation of age deficits. More generally, it is evidence against any explanation that holds that age deficits will always be larger when the task is more difficult. More subtly, we found that while Type III and IV performance was equivalent for young adults, older adults were perhaps slightly better at Type IV than III, providing some additional evidence against a rule-complexity account.

Rabi and Minda (2016) attributed the Type II deficit in older adults to rule-specificity. They argued that older adults were generally unable to learn complex verbal rules. They found very little evidence for perfect correspondence to single-dimensional rules in their data, and supposed that older adults were switching between single-dimensional rules as they received negative feedback on their performance. In our data, we also found evidence against older adults generally being able to learn complex verbal rules in our Hamming distance analysis. Furthermore, we looked closely at the data to see if single-dimensional rule use was plausible.^4^ Our Hamming distance analysis provided additional evidence that single-dimensional rules were not being used consistently by older adults in the Type II task, as they did not appear to be moving closer to single-dimensional rules in that task. Also, our consecutive trial analysis showed that quickly switching between single-dimensional rules did not describe older adults well either. Older adults made responses consistent with using the same single-dimensional rule only on about half of trials in which this behavior could be assessed, even when just looking at pairs of trials in which the first response was correct.

For a rule-based system, using a rule more often after receiving positive feedback on its performance is critical—otherwise no learning is occurring. Stronger assumptions have been made: the COVIS explicit rule-based system assumes that positive feedback always leads to using the same rule again on the next trial (Ashby et al., 1998). As a result, being inconsistent with a single-dimensional rule on half of trials after positive feedback is difficult to explain with a single-dimensional rule system, unless it is working extremely poorly: the system is randomly choosing among all possible rules on each trial with equal probability. However, we show in Appendix B that many participants responded reliably above chance. Together these results make for an argument against older adults using single-dimensional rules in the Type II task, where they showed the greatest deficits.

A remaining possibility for the rule-specificity hypothesis is that older adults were attempting to use multidimensional rules, but were just worse at finding the correct multidimensional rules compared to young adults. Our Hamming distance analysis argued against this interpretation because there was no trend toward older adults moving further away from single-dimensional rules over blocks, but there exist many different proposals of how complex rules are learned (e.g., COVIS, Rational Rules, or Nosofsky, Palmeri, et al.’s, 1994, RULEX) and these would have to be examined in detail.

The clustering hypothesis better accounts for these age deficits. We formalized the clustering hypothesis in the RMC and quantitatively showed that the deficits can be explained as older adults being less able to form new clusters than young adults. The best-fitting RMC showed the expected reversal in Type II and Type IV performance between older and young adults. The RMC also matched the empirical data well on the Hamming distances and consecutive trial analysis that argued against single-dimensional rules.

Using the clustering hypothesis rather than rules to explain age deficits leads to reinterpretations of some past results. For example, Maddox, Pacheco, Reeves, Zhu, and Schnyer (2010) found equal age-related declines in rule-based and information-integration tasks (akin to Type II and Type IV, respectively) when both were generated from four clusters. If older adults struggle to produce as many clusters as young adults, these equal declines would be expected. Additionally, clustering can be used to explain some of the strongest evidence for rule-specificity age deficits: age-related increases in perseverative errors in the WCST (Rhodes, 2004). Clustering models can be used in associative learning tasks to explain how old associations do and do not interfere with new associations: if both old and new associations are part of the same cluster then there will be interference because they cannot be accessed separately, but if old and new associations are part of separate clusters then the new associations can potentially be accessed without interference (e.g., Gershman, Blei, & Niv, 2010). If older adults have more trouble creating new clusters, this then could explain why they show greater perseverative errors when the rule changes in the WCST.

Interpreting age declines as an increased difficulty in constructing new clusters yields a new interpretation of the relationship between working memory capacity and type of task. Rabi and Minda (2016) found that working memory capacity was related to performance on Type II but not on Type I tasks. Instead of interpreting working memory capacity as related to performance on complex rules, we can interpret it as necessary for constructing more clusters, because each additional cluster means that there is more information to represent. Rabi and Minda argued that Type II may be more influenced by working memory than Type IV and recently, Stukken, Van Rensbergen, Vanpaemel, and Storms (2016) showed that higher working memory ability was related to utilization of a greater number of clusters during categorization. However, contrary to this view, Lewandowsky (2011) found that working memory capacity affected performance on Types I–VI similarly. More research is needed to clarify the relationship between working memory and clustering, especially as clustering is more naturally described as implicit memory, and older adults show greater deficits in explicit than implicit memory.

Clustering represents one implicit type of categorization, but there are others. Exemplar models, formalized as the Generalized Context Model (GCM; Nosofsky, 1986) and ALCOVE (Kruschke, 1992), also could potentially explain these results. These exemplar models use the mechanism of selective attention to produce better performance in Type II than Type IV problems. As it is easier to selectively attend to fewer dimensions, Type II has an advantage over Type IV because in Type II one of the dimensions can be completely ignored (see Figure 1). The claim for selective attention has been bolstered by findings that the performance advantage for Type II over Type IV only occurs for separable dimensions, like those used in our experiment, where selective attention can operate. The reverse pattern occurs with integral dimensions, such as the hue and saturation of colors, for which selective attention is much harder to use (Nosofsky & Palmeri, 1996).

A deficit in selective attention is another nonrule-based approach for explaining the reversal of Type II and IV performance between young and older adults. However, it is not clear whether selective attention can explain our results. Maddox, Filoteo, and Huntington (1998) tested selective attention for integral and separable stimuli, investigating how well young and older adults could ignore irrelevant information on nonselected dimensions. They found that for separable dimensions, older adults were just as good as young adults at selective attention, though they only tested application of a known categorization rule rather than learning an unknown rule as we did in our experiment. Future work could combine our task with a selective attention task to see if individual differences in selective attention in older adults correspond with individual differences in Type II and Type IV performance.

In summary, we have demonstrated that utilization of fewer clusters in older adults provides a parsimonious account of age differences in the Shepard et al. (1961) categorization tasks. We argue that this view is more consistent with the data than a rule-complexity account, and a rule-specificity account that postulates a reliance on single-dimensional rules in older adults. This does not mean that older adults do not use single-dimensional rules: although the overall pattern of results was best explained by the RMC with age deficits in both cluster formation and choice, the RMC was not able to match the participant performance on Type I problems, which can be perfectly represented by single-dimensional rules. It could be that a hybrid model that combines single-dimensional rules and a clustering representation would better explain the data, or perhaps a hierarchical elaboration of the RMC that introduces rule-like behavior is needed (Heller, Sanborn, & Chater, 2009). The clustering hypothesis is a start, but there is much about categorization in older adults that still needs investigation.

## Figures and Tables

**Table 1 tbl1:** Model Comparison Between Versions of the Rational Model of Categorization That Tests for Differences in Parameters Between Age Groups

Model	Parameters differing between age groups	Number of parameters	Negative log likelihood	AIC	AIC weights	BIC	BIC weights
1	None	4	20,740	41,488	.0000	41,522	.0000
2	*s*_*P*_	5	20,268	40,547	.0000	40,589	.0000
3	*s*_*L*_	5	20,517	41,044	.0000	41,086	.0000
4	*c*	5	20,383	40,776	.0000	40,819	.0000
5	*r*	5	20,339	40,688	.0000	40,731	.0000
6	*s*_*P*_, *s*_*L*_	6	20,243	40,498	.0000	40,549	.0000
7	*s*_*P*_, *c*	6	20,305	40,622	.0000	40,673	.0000
8	*s*_*P*_, *r*	6	20,221	40,453	.0000	40,504	.0000
9	*s*_*L*_, *c*	6	20,324	40,661	.0000	40,712	.0000
10	*s*_*L*_, *r*	6	20,328	40,668	.0000	40,719	.0000
11	*c, r*	6	20,238	40,489	.0000	40,540	.0000
12	*s*_*P*_, *s*_*L*_, *c*	7	20,241	40,495	.0000	40,555	.0000
13	*s*_*P*_, *s*_*L*_, *r*	7	20,205	40,424	.0000	40,484	.0000
**14**	***s***_***P***_**, *c, r***	**7**	**20,178**	**40,371**	**.7136**	**40,430**	**.9943**
15	*s*_*L*_, *c, r*	7	20,200	40,414	.0000	40,474	.0000
16	*s*_*P*_, *s*_*L*_, *c, r*	8	20,178	40,373	.2864	40,441	.0057
*Note*. *s*_*P*_ is the physical salience parameter, *s*_*L*_ is the label salience parameter, *c* is the coupling parameter, and *r* is the determinism parameter. Negative log likelihood is the goodness of fit of the model (smaller is better), and Akaike’s Information Criterion (AIC) and Bayesian Information Criterion (BIC) are two different measures that balance goodness of fit with a penalty for model complexity (smaller is better). AIC weights and BIC weights transform the AIC and BIC values to approximate the probability of the model given the data (larger is better). The best model by both AIC and BIC is in bold.							

**Table 2 tbl2:** Best-Fitting Rational Model of Categorization Parameters for Young and Older Age Groups

Age	*s*_*P*_	*s*_*L*_	*c*	*r*
Young	0.6888	0.1615	0.5044	0.7738
Older	0.4427	0.1615	0.7450	0.4540
*Note*. *s*_*P*_ is the physical salience parameter, *s*_*L*_ is the label salience parameter, *c* is the coupling parameter, and *r* is the determinism parameter. In the best-fitting model, *s*_*L*_ is the same for young and older age groups.				

**Table 3 tbl3:** Predicted Accuracy for Problem Types I–IV

Parameters	Type I	Type II	Type III	Type IV
Young adults	.86	.77	.70	.73
Older adults choosing like young adults	.87	.55	.64	.69
Older adults	.80	.54	.60	.63

**Table B1 tbl4:** Proportion of Participants Significantly Above Chance in Response Consistency

Age	Type I	Type II	Type III	Type IV
Young	.98	.81	.83	.77
Older	.90	.46	.54	.79

## Footnotes

1 See later for explanation of the use of Bayes factors for comparisons.

2 Throughout this article, any violations of sphericity in ANOVAs were corrected using the Greenhouse-Geisser correction.

3 Type II has been found to be better than Type IV for young adults, but not in every experiment. The effect is more likely to be found under the instructions that we used (Kurtz et al., 2012).

4 We did not fit a model of single-dimensional rule use because there are a variety of ways to implement this approach, so we made a more general qualitative argument.

## Appendix A Details of the Rational Model of Categorization

When making a category judgment, the Rational Model of Categorization (RMC; Anderson, 1991) determines the probability that a new item, item *n*, belongs to the *i*th category (*y*_*n*_ *= i*), based on the physical features of that item, *x*_*n*_, the physical features of all of the previously seen items, **x_n−1_**, and the category labels of all of the previously seen items, **y_n−1_**. This can be written as the probability of the category label and physical features of the new item, given all the previously seen items and labels

$$P\left(y_{n}=i|x_{n},{\text{x}}_{\text{n-1}},{\text{y}}_{\text{n-1}}\right)\propto P\left(y_{n}=i,x_{n}|{\text{x}}_{\text{n-1}},{\text{y}}_{\text{n-1}}\right).$$

This probability is then transformed into the probability of making a binary category response *i* by raising it to the exponent *r* and then renormalizing

$$\frac{P{\left(y_{n}=i|x_{n},{\text{x}}_{\text{n-1}},{\text{c}}_{\text{n-1}}\right)}^{r}}{P{\left(y_{n}=i|x_{n},{\text{x}}_{\text{n-1}},{\text{c}}_{\text{n-1}}\right)}^{r}+{\left(1-P\left(y_{n}=i|x_{n},{\text{x}}_{\text{n-1}},{\text{c}}_{\text{n-1}}\right)\right)}^{r}}.$$

The probability of a new item and its category label is a weighted sum of the probability of the item and label arising from each of the clusters. Because the cluster indices are inferred from the data, these are marginalized out

$$P\left(y_{n}=i,x_{n}|{\text{x}}_{\text{n-1}},{\text{y}}_{\text{n-1}}\right)=\underset{{\text{z}}_{\text{n}}}{\sum }P\left(y_{n}=i,x_{n}|{\text{z}}_{\text{n}},{\text{x}}_{\text{n-1}},{\text{y}}_{\text{n-1}}\right)P\left({\text{z}}_{\text{n-1}}|{\text{x}}_{\text{n-1}},{\text{y}}_{\text{n-1}}\right)P(z_{n}|{\text{z}}_{\text{n-1}}),$$ A1

where *z*_*n*_ is the cluster index of the cluster for item *n*, and **z_n-1_** are the cluster indices for the previously seen items.

There are three terms on the right-hand side of Equation A1 that must be defined. First, the prior probability of the cluster index of the new item *P*(*z*_*n*_|**z**_**n−1**_) is defined as a Chinese Restaurant Process prior that can flexibly interpolate between a single cluster for all the items and a different cluster for each item (Aldous, 1985). This prior can be written as a simple sequential rich-get-richer process

$$P\left(z_{n}=k|{\text{z}}_{\text{n-1}}\right)=\left\{\begin{array}{c} \frac{cM_{k}}{\left(1-c\right)+c\left(i-1\right)}\text{ if}\;M_{k}>0\;\left(\text{i}\text{.e}\text{.,}\;k\;\text{is}\;\text{old}\right)\\ \frac{1-c}{\left(1-c\right)+c\left(i-1\right)}\text{ if}\;M_{k}=0\;\left(\text{i}\text{.e}\text{.,}\;k\;\text{is}\;\text{new}\right) \end{array}\right\}$$

where *M*_*k*_ is the number of items assigned to cluster *k* and *c* is the coupling parameter that helps determine the number of clusters.

Second, the posterior on cluster assignments for previous items can also be built up as a sequential process

$$P\left({\text{z}}_{\text{n-1}}{\text{|x}}_{\text{n-1}}{\text{,y}}_{\text{n-1}}\right)\propto P\left(y_{n-1},x_{n-1}|{\text{z}}_{\text{n-1}},{\text{x}}_{\text{n-2}},{\text{y}}_{\text{n-2}}\right)P\left({\text{z}}_{\text{n-2}}|{\text{x}}_{\text{n-2}},{\text{y}}_{\text{n-2}}\right)P(z_{n-1}|{\text{z}}_{\text{n-2}}).$$ (A2)

We used the original inference algorithm for the RMC which assigns an item to the cluster that had the highest probability of producing that item. This approximation makes simulation feasible relative to summing Equation A1 over all possible partitions of the items into clusters, and it is deterministic which improves speed of computation. This approximation is sensitive to the ordering of the items, so the likelihood of each participant’s responses was computed given the order in which he or she saw the items.

Finally, we need the likelihood of an item and its category label given the other items that are already assigned to a cluster. Because the RMC assumes that within a cluster the category label is independent from the physical features, and that all of the physical features are independent from one another

$$P\left(y_{n}=i,x_{n}|{\text{z}}_{\text{n}},{\text{x}}_{\text{n-1}},{\text{y}}_{\text{n-1}}\right)=P\left(y_{n}=i|{\text{z}}_{\text{n}},{\text{y}}_{\text{n-1}}\right)\underset{j}{\prod }P(x_{j,n}|{\text{z}}_{\text{n}},{\text{x}}_{\text{n-1}}),$$

where *x*_*j,n*_ is the value of the *j*th physical feature of item *n*.

The Shepard et al. (1961) learning task used three binary-valued physical features, so a Beta-binomial likelihood distribution was used

$$P\left(x_{j,n}=v|{\text{z}}_{\text{n}},{\text{x}}_{\text{n-1}}\right)=\frac{B_{v}+s_{P}}{B_{.}+2s_{P}},$$

where *B*_*v*_ is the number of items in the cluster that match the new item along the *j*th physical feature, *B*. is the number of items in the cluster, and *s_P_* is the parameter of a symmetric Beta prior distribution on the probability of obtaining different features. The likelihood of the binary-valued category feature uses the same form with a separate symmetric Beta prior

$$P\left(y_{n}=v|{\text{z}}_{\text{n}},{\text{y}}_{\text{n-1}}\right)=\frac{B_{v}+s_{L}}{B_{.}+2s_{L}}.$$

The parameters used to infer the category label and those used to infer the cluster assignments are defined by the model to be the same, but it is possible to use separate parameters in the two operations (e.g., Nosofsky, 1991). In the results, we separated the two processes by computing Equation A2 with one set of parameters, and then computing the response probabilities in Equation A1 (given the indices computed in Equation A2) using a different set of parameters.

### Model Fitting Details

The likelihood of participant responses was determined using a Bernoulli likelihood. We implemented our model in R and used the nlm function from the stats package (R Core Team, 2016) to search for the best-fitting parameters. The parameter search was difficult because the predictions of the RMC can change suddenly with small changes in the parameters (e.g., Sanborn, Griffiths, & Navarro, 2010). We eventually settled on a procedure of running the nlm function until it converged and then restarting the algorithm by randomly jittering the best-fitting parameters. The random new starting point was a sample from a Gaussian distribution centered on the previous best fit with a *SD* of 0.5% of the value of the parameter. We restarted the algorithm at least 30 times for each model, but finding that some of the models were still improving, we repeated this exercise until the log likelihood appeared stable and all models nested within more general models fit worse than the more general models. For the collection of models we analyzed, the parameter search required a month on a desktop computer.

## Appendix B Response Consistency

The experimental results showed that for older adults in the Type II task their responses matched between maximally different stimuli on approximately half of trials, even when they were correct on the previous trial. This pattern of behavior could be a result of random responding, or equivalently the result of choosing a new single-dimensional rule with equal probability on each trial from among the complete set of single-dimensional rules.

To determine if young or older adults were randomly responding in any of the tasks, we calculated a measure of response consistency, which can identify participants who are at chance accuracy but are still making consistent responses. This measure calculated for each stimulus the proportion of trials on which participants made the same response, which ranged from 0.5 (i.e., Alpha to half the trials and Beta to the other half) to 1 (i.e., responses were either always Alpha or always Beta).

We simulated the distribution of response consistency for individuals performing according to chance. Ordering the simulated response consistency from lowest to highest, the 95th percentile was almost exactly 2/3, so this value was used as the cut-off for significance. The proportion of participants that were significantly above chance in response consistency is shown in Table B1 for each age group and task type.[Table-anchor tbl4]
